# Supplementary material for: A Variable Neighbourhood Descent Heuristic for Conformational Search Using a Quantum Annealer
Source: Sci Rep. 2019 Sep 23;9:13708. doi: 10.1038/s41598-019-47298-y (PMC6757033; doi:10.1038/s41598-019-47298-y)
Supplement: Supplementary file 1 — Supplementary Information [file 41598_2019_47298_MOESM1_ESM.pdf]

Supplementary Information
Article in Scientific Reports

A Variable Neighbourhood Descent Heuristic for Conformational Search Using a Quantum Annealer

D. J. J. Marchand[1], M. Noori[1,*], A. Roberts[1], G. Rosenberg[1], B. Woods[1], U. Yildiz[1], M. Coons[2], D. Devore[2], and P. Margl[2]

[1]1QB Information Technologies (1QBit), 458-550 Burrard Street, Vancouver, BC, Canada, V6C 2B5
[2]The Dow Chemical Company, Core R&D, 1776 Building, Midland, MI, United States, 48674
[*]moslem.noori@1qbit.com
Names listed by affiliation and in alphabetical order.

A Brief Overview of Quantum Annealing

The D-Wave 2000Q quantum annealer solves problems in the form of an Ising Hamiltonian defined by

$$\begin{aligned} \arg\min_{\mathbf{s}} \quad & \mathbf{s}^T\mathbf{J}\mathbf{s}+\mathbf{h}^T\mathbf{s}, \\ \text{s.t.} \quad & \mathbf{s}\in\{-1,1\}^N, \end{aligned}$$

where N is the number of qubits and $\mathbf{h}$ and $\mathbf{J}$ represent the local fields and couplers, respectively. The above Ising problem can be transformed into a quadratic unconstrained binary optimization (QUBO) problem

$$\begin{aligned} \min_{\mathbf{x}} \quad & \mathbf{x}^T\mathbf{Q}\mathbf{x}, \\ \text{s.t.} \quad & \mathbf{x}\in\{0,1\}^N \end{aligned}$$

by substituting $\mathbf{s}=2\mathbf{x}-\mathbf{1}$. To utilize the quantum annealer, the local fields and the pairwise couplings of the Ising formulation ($\mathbf{h}$ and $\mathbf{J}$) must be specified.

Quantum annealing is inspired by the adiabatic principle: a system at equilibrium, in its ground state, will remain in the ground state provided the system is evolved sufficiently slowly. During each annealing cycle, the Hamiltonian of the system is continuously deformed from a known, simple Hamiltonian H_0 to the problem Hamiltonian H_{Ising}, which is typically a non-trivial task. This evolution is represented by the Hamiltonian

$$H(\tau)=A(\tau)H_0+B(\tau)H_{\text{Ising}}, \quad \tau\in[0,1], \tag{1}$$

where $\tau=t/t_a$, with t_a representing the annealing time. The functions $A(\tau)$ and $B(\tau)$ specify the annealing schedule. Typically, $A(\tau)$ and $B(\tau)$ are monotonically decreasing and increasing functions, respectively.

Given that the quantum annealer operates at a very low but finite temperature, the quantum annealer is a heuristic solver. For this reason, typical use of the quantum annealer involves obtaining an ensemble that consists of the results of hundreds, if not thousands, of reads from the quantum annealer. Additional specifications of the quantum annealer include the annealing time, the scaling of the **h** and **J** values, and using gauges to compensate for errors due to noise. We refer the interested reader to[1] for more details on quantum annealers and their applications.

Choice of Molecules

Source (Experimental) Structures

For systems A–C in Fig. 4 of the paper, atomic coordinates from single-crystal X-ray structures are available, either for the compounds themselves (B and C)[2–4] or for close analogues (A)[5]. Systems A and C represent the active, cationic (A) and inactive, initial form (C) of two olefin polymerization catalysts. System B embodies a catalyst for olefin metathesis. X-ray experimental data pertaining to the three-dimensional (3D) structures of ortho-phenylenes (oPh) are currently confined to substituted analogues up to chain lengths of ten phenylene units[6]; the longest-chain unsubstituted oPh^x instance is for $x = 5$[2,7,8]. Complementary computational studies exist for $x = 12$ and support the experimental 3D structures[9]. Experiments and computation agree that the preferred morphology for oPh^x oligomers is the tightly helical, "closed-helix" conformation. In the following, we refer to the geometries that correspond directly to experiments (or high-level computation for system A) as *source* geometries.

Initial Conformations

In the present work, we have focused exclusively on the torsional degrees of freedom, while other molecular degrees of freedom such as the bond lengths and angles were fixed at the values they assumed in the *initial* structure and excluded from the optimization. We employed two strategies to create initial structures. The details of these methods are given below; the geometries resulting from them are referred to as initial geometries in the following.

- **Method a:** Guesses pertaining to the structure of metal compounds were created using off-the-shelf molecular modelling software based on the structure of experimentally characterized analogues (compound A)[5,10,11] or directly obtained from the X-ray diffraction structures (compounds B and C)[2,4,12]. These structures were locally optimized using the ORCA 4 program[13,14] in order to remove minor inconsistencies in the atomic positions. For this, we used the popular M06 density functional[15] and a def2-SVP basis set[16], along with the corresponding fit basis set on non-metal atoms and a def2-TZVP basis set[17] on metal atoms, together with the appropriate ECPs[18]. The RI-JK[19] and RIJCOSX[20] approximations were employed for acceleration. An unrestricted wave function with four unpaired spins was used for system C.

- **Method b:** Ortho-phenylene (oPh) geometries were generated as closed helices[9] using Materials Studio[21] by optimizing them with the Universal force field[22] implemented in Materials Studio. There is evidence that the open and closed helical states of oPh^n are close in energy[9] to the majority of experimental evidence gathered in the solid state pointing to a general preference for the closed helix[6,23–26]. Using UFF to optimize all bond lengths, angles, and torsions, we found that the closed and open helix conformers of oPh^{20} differ by 1.5 kcal/mol per phenylene unit, favouring the closed helix.

From Initial Conformations to Reference Conformations

Reference conformations for all nine molecules, embodying a best-effort guess of the global minima available to the *initial* conformations in the torsional space, were generated using an implementation of the parallel tempering Monte Carlo (PTMC) method. All other experiments described in the paper were stopped once a conformation with an energy within 0.1 kcal/mol of the energy of the reference conformation was found.

The agreement between *source* structures, which closely mirror or are identical to experimental structures, and reference structures found by PTMC is significant for several reasons. Source structures embody the best available information regarding the global minimum geometry of the system. In order to be useful, a conformational search algorithm should be able to locate this geometry starting without the use of prior knowledge of the optimal set of torsion angles. The reference structure, therefore, should closely approximate the source structure. If they do not agree, several causes may be contributing to the disagreement:

- **Force field failure:** The force field guiding the search of the torsional energy surface may be insufficiently realistic to capture the true global minimum.
- **Structural bias:** The initial set of bond lengths and bond angles supplied with the initial structure may bias the conformational search in the torsional space toward regions inconsistent with the source geometry.
- **Experimental bias:** The source geometry itself may be dominated by effects not present in an isolated, molecular system, as might be the case if the source geometry is taken from a crystal with significant intermolecular interactions.

Table 1 summarizes the differences between source and reference geometries. Agreement between the reference structures and the source structures is indicated by the contents of the “Reference Matches Source" column. In general, reference structures correspond closely to the source structures; except for the case of system B, source and reference structures occupy the same energy basin. For system B, the PTMC search identified a basin with a minimum energy lower than the source structure by ≈ 1.5 kcal/mol. This deviation is attributable either to force-field failure (due to the use of a simplified Universal force field (UFF) during PTMC) or experimental bias (see above) due to subtle effects of crystal packing on the source geometry.

Model system	Type	Torsions	Source	Method	Reference matches source
A	metal comp’d	6	TW	a	Y
B	metal comp’d	9	[2,3]	a	N
C	metal comp’d	14	[2,4]	a	Y
D	n-alkane	7	[27]	b	Y
E	n-alkane	12	[27]	b	Y
F	n-alkane	17	[27]	b	N
G	o-phenylene	9	TW	b	Y
H	o-phenylene	15	TW	b	Y
I	o-phenylene	19	TW	b	Y

Table 1. Geometric properties of model systems A–I. “Model system” gives the label assigned to each model, “Type” is the type of the model system, “Torsions” is the number of free rotating torsion bonds, “Source” is the source for the initial conformation (where “TW” stands for “this work”), “Method” is the method for optimizing the source conformation to yield the initial conformation, and “Reference matches source" indicates whether the source and reference conformations occupy the same energy basin.

Lowest-Energy Conformations Found by VND

Table 1 and Table 2 of the Results section of the paper present energy residuals of the conformers produced by VND. To provide a better understanding of the quality of the conformers, Table 2 presents the root mean square (RMS) distance between the torsion vector of the reference conformer and the best conformer found by both VND used with the quantum annealer and VND used with an exact solver. The latter method provides an estimate of the potential gains of improvements to the hardware. We see that in this case the best conformers are very close to the reference conformers and clearly in the same basin. On the other hand, the results of VND when using the quantum annealer have larger RMS distances. One reason there are larger RMS distances when using the quantum annealer even for small residuals is symmetries. Taking model system A, for example, there are conformations of the tertiary butyl group that are rotated by 60 degrees, a change that does not have a large impact on the energy. Due to these symmetries, RMS distances are of limited usefulness when comparing molecular geometries. To address this limitation, we present graphical representations of the lowest-energy conformations found for model systems B, F, and I shown as an overlay on the reference conformations (see Fig. 1, Fig. 2, and Fig. 3). We also provide a file with all initial, reference, and optimized molecular conformations.

Model system	VND and quantum annealer	VND and exact solver
A	73.12°	0.41°
B	10.54°	0.47°
C	89.24°	1.22°
D	2.29°	0.91°
E	1.46°	1.07°
F	2.06°	0.00°
G	78.20°	2.03°
H	114.86°	5.96°
I	84.56°	3.96°

Table 2. Root mean square distance between the torsion vector of the reference conformation and the best conformation found with the VND method using the quantum annealer and the VND method using exact solver.

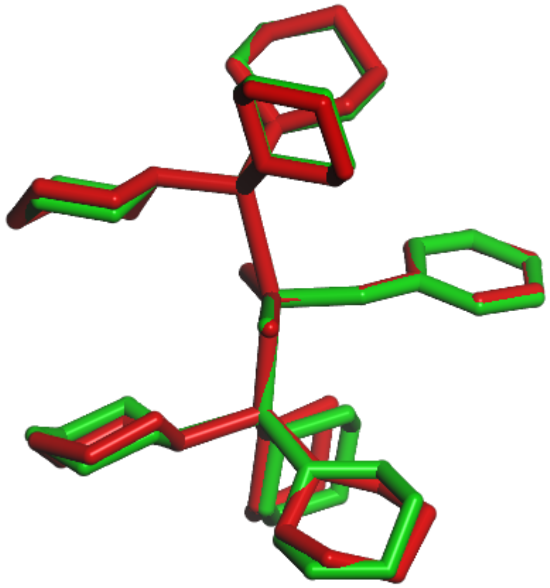

Figure 1. Overlay of the reference conformation (green) and the best conformation found by the VND method using a quantum annealer (red) for model system B.

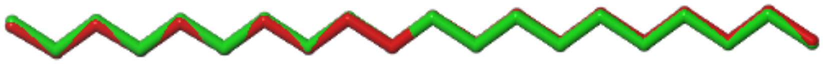

Figure 2. Overlay of the reference conformation (green) and the best conformation found by the VND method using a quantum annealer (red) for model system F.

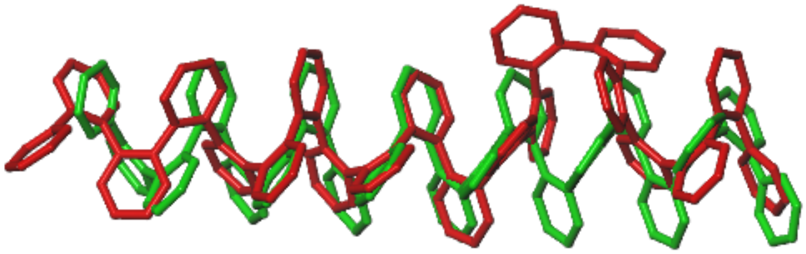

Figure 3. Overlay of the reference conformation (green) and the best conformation found by the VND method using a quantum annealer (red) for model system I.

Further Details of the Experiments

Parameters of different methods for generating the experimental results are explained in the following.

- **PTMC**: Our implementation of PTMC performs 10 parallel MC experiments (replicas) at different temperatures. Each sweep of PTMC consists of proposing M moves, one for each torsion, for each of the replicas (i.e., a total of $10M$ proposals to sweep over all replicas). The proposed moves are accepted according to the Metropolis criterion. Initial temperatures of the replicas are distributed geometrically, given the temperatures of the first and last replicas. The temperatures are automatically updated during the experiment to maintain reasonable Monte Carlo and replica exchange acceptance probabilities.
- **D-Wave 2000Q**: To solve each of the QUBO problems created for optimizing a subset of torsions, a total of 5000 solutions (reads) from the the quantum annealer are obtained and the best solution from among them is used. To improve the results obtained from the annealer, 10 gauges are applied, meaning that the 5000 solutions are obtained by making 10 calls, each with a different gauge, to the quantum annealer, where each call returns 500 solutions. The annealing time is 5 microseconds for each of the reads. Using a deterministic embedding solver that finds complete embeddings with chains of equal length[28], the largest complete graph that can be embedded on the quantum annealer has a size of 63. Majority voting is used to settle the disagreement between the qubits of the chain assigned to a logical binary variable.
- **VND**: The VND results are obtained for $b_{\text{max}} = 200$ and $b_{\text{nc}} = 10$. To respect the size of the largest complete graph embeddable onto the quantum annealer's hardware graph, we set $s = 63$.

Notes on Quantum Annealer Parameter Tuning

There are several parameters affecting the performance of the D-Wave 2000Q quantum annealer in its solving of QUBO problems. Some, such as the connectivity of the qubits and the intrinsic control error (ICE), are the specifications of the hardware over which the user does not have control, while other parameters are tunable. Among those that are tunable, we focused on the annealing time, the effect of having different numbers of gauges, the number of reads (i.e., the solutions obtained from the quantum annealer for the same Ising problem), and the strength of the couplings between the qubits in a chain assigned to a logical bit (here called the *scale*). Further, we explored the effect of the penalty value used for incorporating the one-hot encoding constraints on the quality of solutions.

To tune the above parameters, we generated a small number of QUBO problems for each molecule, created at different iterations of the VND method, and used them as test cases for evaluating the parameter settings. To assess the performance of each set of parameters, we considered the best solution and the percentage of solutions that were

feasible (i.e., those that satisfy all one-hot encoding constraints) within a given number of reads from the quantum annealer. Our tuning effort resulted in the choice of parameter settings introduced in the previous section.

We note that the selected set of families of molecules have distinct properties based on the QUBO coefficients and the connectivity of the variables generated during iterations of VND. Our parameter tuning strategy may result in obtaining a set of parameters that perform relatively well for all families of molecules taken together, but may not necessarily be the best for each of them separately. Thus, we expect that a family-based parameter tuning could yield improved results. This can be achieved by performing a greater number of tuning iterations, or by considering a broader set of tunable quantum annealer parameters, both requiring additional quantum annealing time.

References

1. Biswas, R. *et al.* A NASA perspective on quantum computing: Opportunities and challenges. *Parallel Comput.* **64**, 81–98 (2017).
2. Groom, C. R., Bruno, I. J., Lightfoot, M. P. & Ward, S. C. The Cambridge Structural Database. *Acta Crystallogr. B* 171–179 (2016).
3. Torker, S., Müller, A., Sigrist, R. & Chen, P. Tuning the Steric Properties of a Metathesis Catalyst for Copolymerization of Norbornene and Cyclooctene toward Complete Alternation. *Organometallics* **29**, 2735–2751 (2010).
4. Kawakami, T., Ito, S. & Nozaki, K. CCDC 1416774: Experimental crystal structure determination (2015).
5. Terao, H. *et al.* Phenoxycycloalkylimine ligated zirconium complexes for ethylene polymerization: Formation of vinyl-terminated low molecular weight polyethylenes with high efficiency. *Macromolecules* **39**, 8584–8593 (2006).
6. Mathew, S., Crandall, L. A., Ziegler, C. J. & Hartley, C. S. Enhanced helical folding of ortho-phenylenes through the control of aromatic stacking interactions. *J. Am. Chem. Soc.* **136**, 16666–16675 (2014).
7. Gomes, L., Santos, l. & Low, J. CSD communication (2012).
8. Gomes, L., Santos, l. & Low, J. CCDC 808554: Experimental crystal structure determination (2014).
9. He, J. *et al.* Ortho-Phenylenes: Unusual conjugated oligomers with a surprisingly long effective conjugation length. *J. Am. Chem. Soc.* **132**, 13848–13857 (2010).
10. Terao, H. *et al.* CCDC 635962: Experimental crystal structure determination (2014).
11. Terao, H. *et al.* CCDC 635963: Experimental crystal structure determination (2014).
12. Torker, S., Muller, A., Sigrist, R. & Chen, P. CCDC 811503: Experimental crystal structure determination (2014).
13. Neese, F. The ORCA program system. *Wiley Interdiscip. Rev. Comput. Mol. Sci.* **2**, 73–78.
14. FAccTs GmbH. ORCA 4.0.1 (2017).
15. Zhao, Y. & Truhlar, D. G. The M06 suite of density functionals for main group thermochemistry, thermochemical kinetics, noncovalent interactions, excited states, and transition elements: Two new functionals and systematic testing of four M06-class functionals and 12 other function. *Theor. Chem. Accounts* **120**, 215–241 (2008).
16. Weigend, F. & Ahlrichs, R. Balanced basis sets of split valence, triple zeta valence and quadruple zeta valence quality for H to Rn: Design and assessment of accuracy. *Phys. Chem. Chem. Phys.* 3297–3305.
17. Weigend, F. Accurate Coulomb-fitting basis sets for H to Rn. *Phys. Chem. Chem. Phys.* **8**, 1057–1065 (2006).
18. Andrae, D., Häußermann, U., Dolg, M., Stoll, H. & Preuß, H. Energy-adjustedab initio pseudopotentials for the second and third row transition elements. *Theor. Chimica Acta* **77**, 123–141 (1990).
19. Weigend, F. A fully direct RI-HF algorithm: Implementation, optimised auxiliary basis sets, demonstration of accuracy and efficiency. *Phys. Chem. Chem. Phys.* **4**, 4285–4291 (2002).
20. Neese, F., Wennmohs, F., Hansen, A. & Becker, U. Efficient, approximate and parallel Hartree-Fock and hybrid DFT calculations. A 'chain-of-spheres' algorithm for the Hartree-Fock exchange. *Chem. Phys.* **356**, 98–109 (2009).
21. Dassault Systemes BIOVIA. Materials Studio 2016 (2017).

22. Rappé, A. K., Casewit, C. J., Colwell, K. S., Goddard, W. A. & Skiff, W. M. UFF, a Full Periodic Table Force Field for Molecular Mechanics and Molecular Dynamics Simulations. *J. Am. Chem. Soc.* **114**, 10024–10035 (1992).

23. Sato, H. *et al.* Redox-responsive molecular helices with highly condensed π-clouds. *Nat. Chem.* **3**, 68 (2011).

24. Blake, A. J., Cooke, P. A., Doyle, K. J., Gair, S. & Simpkins, N. S. Poly-orthophenylenes: Synthesis by Suzuki coupling and solid state helical structures. *Tetrahedron Lett.* **39**, 9093–9096 (1998).

25. Mathew, S. M., Engle, J. T., Ziegler, C. J. & Hartley, C. S. The role of arene-arene interactions in the folding of ortho-phenylenes. *J. Am. Chem. Soc.* **135**, 6714–6722 (2013).

26. Mathew, S. M., Engle, J. T., Ziegler, C. J. & Hartley, C. S. CCDC 954742: Experimental crystal structure determination (2014).

27. The last globally stable extended alkane. *Angewandte Chemie, Int. Ed.* **52**, 463–466 (2013).

28. Boothby, T., King, A. D. & Roy, A. Fast clique minor generation in chimera qubit connectivity graphs. *Quantum Inf. Process.* **15**, 495–508 (2016).
